# The Cooperative Effect of Genistein and Protein Hydrolysates on the Proliferation and Survival of Osteoblastic Cells (hFOB 1.19)

**DOI:** 10.3390/molecules21111489

**Published:** 2016-11-08

**Authors:** Shuo Wang, Yu Fu, Xin-Huai Zhao

**Affiliations:** 1 Key Laboratory of Dairy Science, Ministry of Education, Northeast Agricultural University, 150030 Harbin, China; wangshuo920102@163.com (S.W.); yu.fu@food.au.dk (Y.F.); 2 Department of Food Science, Northeast Agricultural University, 150030 Harbin, China; 3 Department of Food Science, Aarhus University, Blichers Allé 20, 8830 Tjele, Denmark

**Keywords:** genistein, protein hydrolysates, osteoblasts, proliferation, anti-apoptosis

## Abstract

Chum salmon skin gelatin, de-isoflavoned soy protein, and casein were hydrolyzed at two degrees of hydrolysis. Genistein, the prepared hydrolysates, and genistein-hydrolysate combinations were assessed for their proliferative and anti-apoptotic effects on human osteoblasts (hFOB 1.19) to clarify potential cooperative effects between genistein and these hydrolysates in these two activities. Genistein at 2.5 μg/L demonstrated the highest proliferative activity, while the higher dose of genistein inhibited cell growth. All hydrolysates promoted osteoblast proliferation by increasing cell viability to 102.9%–131.1%. Regarding etoposide- or NaF-induced osteoblast apoptosis, these hydrolysates at 0.05 g/L showed both preventive and therapeutic effects against apoptosis. In the mode of apoptotic prevention, the hydrolysates decreased apoptotic cells from 32.9% to 15.2%–23.7% (etoposide treatment) or from 23.6% to 14.3%–19.6% (NaF treatment). In the mode of apoptotic rescue, the hydrolysates lessened the extent of apoptotic cells from 15.9% to 13.0%–15.3% (etoposide treatment) or from 13.3% to 10.9%–12.7% (NaF treatment). Gelatin hydrolysates showed the highest activities among all hydrolysates in all cases. All investigated combinations (especially the genistein-gelatin hydrolysate combination) had stronger proliferation, apoptotic prevention, and rescue than genistein itself or their counterpart hydrolysates alone, suggesting that genistein cooperated with these hydrolysates, rendering greater activities in osteoblast proliferation and anti-apoptosis.

## 1. Introduction

Osteoporosis as a systemic skeletal disease is one of the common health problems affecting a large number of people around the world. Osteoporosis is characterized by micro-architectural deterioration of the bone tissue and lower bone mass, with the consequent increase in bone fragility and fracture susceptibility [1]. Bone is a complicated tissue with osteoblasts, osteoclasts, osteocytes, and lining cells [2]. Bone remodeling and turnover are regulated via respective bone formation and resorption by respective osteoblasts and osteoclasts [3]. The imbalance between bone formation and resorption, thus, causes osteoporosis [3]. Traditional therapeutic agents for osteoporosis include calcium and extra vitamin D (V_D_), estrogen, bisphosphonates, etc. However, these agents usually result in some side effects on the body; for example, it has been reported that hormone therapy may increase the risk of breast and endometrial cancers [4]. It is very essential in the present to develop functional foods and food ingredients with positive impacts on bone health but without these undesired side effects.

Soy, one of the important agricultural commodities, contains phytoestrogens with potential healthcare effects on osteoporosis in postmenopausal women [5]. Soy-contained isoflavones are able to increase mineral density of spine bone significantly [5], inhibit bone resorption, stimulate bone formation [6,7], promote osteoblast proliferation, inhibit osteoclast proliferation, and increase osteoblast mineralized nodules [8]. Genistein, as one main member of the isoflavone family, can increase proliferation of human bone marrow mesenchymal stem cells in a time- and dose-dependent manner [9]. Both in vitro and in vivo studies have proposed that isoflavones are potential alternatives for hormone replacement therapy.

Protein hydrolysates as food supplements can exhibit beneficial effects on bone metabolism [10,11,12]. Casein phosphopeptides can directly influence the growth of osteoblastic cells (MC3T3-E1) [13], stimulate calcium absorption, and affect the differentiation of human primary osteoblast-like cells [14]. Soy protein isolate exerts healthcare functions on the bone through increasing bone volume fraction, bone mineral density, connectivity density, and other effects [15], and can prevent high fat diet-induced bone impairments [16]. Soy protein hydrolysates are able to reduce bone turnover in postmenopausal women [17]. Collagen hydrolysate is a potential therapeutic ingredient for osteoporosis [11]. They can improve bone metabolism, decrease osteoclast activity [18], and enhance organic substance content of the bone [19]. In addition, several recent studies have revealed the in vivo biological activity of collagen and gelatin peptides to bone health [20,21,22]. Chum salmon skin gelatin hydrolysates can enhance osteoblast proliferation and antagonize NaF-induced osteoblast apoptosis [23]. Gelatin hydrolysate can offer high levels of some amino acids, among which are glycine and proline that play a pivotal role in the synthesis type I collagen. Type I collagen, as a key component, accounts for 95% of bone proteins [24,25]. A recent study also reveals that both casein and soy protein hydrolysates have estradiol-like action on human fetal osteoblasts (hFOB 1.19 cells) by promoting cell growth [26].

Isoflavones, proteins, and protein hydrolysates are potential ingredients of daily foods and, especially, functional foods. Protein digestion in the body will generate protein hydrolysates composed of various peptides. Thus, protein hydrolysates may have an opportunity to interact with isoflavones in the body. However, it is unknown that if isoflavones can cooperate with protein hydrolysates to render enhanced or decreased activities in osteoblasts. The hFOB 1.19 cells, as a homogenous and rapidly-proliferating model, are normally investigated for human osteoblast differentiation, physiology, and impacts of cytokines on the function of osteoblasts [27,28]. Osteoblast apoptosis plays a pivotal role in maintenance and development of bone [29]. It is documented that 60%–80% of osteoblasts assembled at the resorptive pit die through apoptosis [30]. In addition, bone loss induced by deficient sex steroid, excessive glucocorticoid, or aging, is partially attributed to osteoblast apoptosis [31]. Although different hormones and anti-osteoporotic drugs exert beneficial effects on the bone by inhibiting osteoblast apoptosis, the issue of whether the cooperative effect of genistein and protein hydrolysates on proliferation and apoptosis of the hFOB 1.19 cells remains to be explored. Therefore, the aim of this study was to verify synergistic effects between genistein and these hydrolysates on osteoblast proliferation and anti-apoptosis. In this study, genistein was selected and combined with three protein hydrolysates, which were previously derived from chum salmon skin gelatin, de-isoflavoned soy protein, and casein using papain. Genistein, these hydrolysates, and genistein-hydrolysate combinations were assessed and compared for their in vitro activities to the hFOB 1.19 cells. Based on our previous studies [23,26], cell viability was used to examine osteoblast proliferation, while etoposide (EP) and NaF (apoptosis inducers) were used to induce osteoblast apoptosis.

## 2. Results

### 2.1. Degrees of Hydrolysis and Amino Acid Compositions of the Prepared Hydrolysates

In this study, three proteins (gelatin, de-isoflavoned soy protein, and casein) were hydrolyzed by papain of 3 kU/g protein at two degrees of hydrolysis (DH). Gelatin was hydrolyzed for 2 and 7 h to generate GH1 and GH2 with DH values of 7.7% and 13.7%, respectively. De-isoflavoned soy protein was hydrolyzed for 3 and 8 h to obtain SH1 and SH2 with DH values of 7.6% and 13.5%, respectively. Casein was hydrolyzed for 1.5 and 7 h to prepare CH1 and CH2 with DH values of 7.3% and 13.3%, respectively.

Amino acid compositions of GH2, SH2, and CH2 were analyzed, and the results are listed in Table 1. The data indicate that three hydrolysates had different amino acid profiles. GH2 contained 4-Hyp of 110.7 g/kg protein; however, 4-Hyp was not detected in both SH2 and CH2. Moreover, GH2 was also rich in three amino acids (Ala, Gly, and Pro) than SH2 and CH2. These different amino acid profiles might confer three hydrolysates with different activities in the studied osteoblasts. How the amino acid profile governed the activities of these protein hydrolysates was out of the aim of this study and, therefore, is not discussed here. However, this issue is important and, thus, needs a future investigation.

### 2.2. Growth Proliferation and Inhibition of Genistein on the Osteoblasts

**Figure 1:**
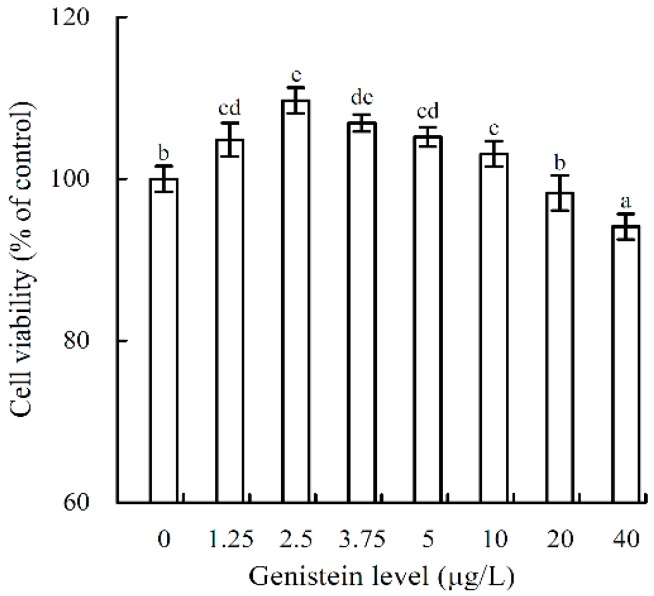
Cell viability of osteoblasts treated with genistein at 0–40 μg/L for 48 h. The values are presented as means ± standard deviations (*n* = 3). Different letters indicate significantly different values (*p* < 0.05) using one-way ANOVA analysis.

The results showed that genistein at 1.25–40 μg/L had both proliferative and inhibitory activities in the osteoblasts (Figure 1). When the osteoblasts were incubated with genistein for 48 h, the measured values of cell viability ranged from 94.1% to 109.7%. Genistein at doses of 1.25–10 μg/L resulted in weaker proliferation in the osteoblasts, as measured values of cell viability were all higher than 100%. Genistein at a dose of 2.5 μg/L had the highest osteoblast proliferative activity, reflected by the highest value of 109.7% (*p* < 0.05). However, genistein at doses of 20–40 μg/L exhibited pronounced growth inhibition in osteoblasts, as the measured values of cell viability decreased to 94.1%–98.3%. In other words, a higher dose of genistein was toxic to the osteoblasts. Therefore, genistein at a dose of 2.5 μg/L was used in later evaluations.

### 2.3. Cooperation between Genistein and the Hydrolysates in Osteoblast Proliferation

When the osteoblasts were treated with genistein, the hydrolysates, and genistein-hydrolysate combinations for 48 h, the detected values of cell viability are listed in Table 2. The six hydrolysates at dose levels of 0.02–0.1 g/L could promote cell growth, increasing the values of cell viability to 108.4%–131.1% (GH1 and GH2), 104.6%–121.3% (SH1 and SH2), and 102.9%–112.4% (CH1 and CH2). The positive control (17β-estradiol) could also promote cell growth (cell viability of 134.2%). The data also indicated that the hydrolysates with higher DH values always led to higher proliferative activities than the counterpart hydrolysates with lower DH values, and gelatin hydrolysates showed the strongest activities in the osteoblasts than other hydrolysates with similar DH values. The genistein-GH1 (or genistein-GH2) combination enhanced cell viability to 113.8%–127.2% (or 125.3%–140.9%). The genistein-SH1 (or genistein-SH2) combination resulted in cell viability of 104.8%–114.6% (or 119.6%–123.1%). The genistein-CH1 (or genistein-CH2) combination led to cell viability of 101.9%–110.5% (or 106.7%–114.6%). These results demonstrated that employment of genistein (2.5 μg/L) together with one of the six hydrolysates (0.02–0.1 g/L) resulted in higher cell viability than genistein itself, or counterpart hydrolysates alone. It is suggested that a cooperative effect may exist between genistein and the hydrolysates in osteoblast proliferation. In other words, genistein cooperated well with these hydrolysates (especially GH1 and GH2), contributing to stronger activity in promoting osteoblast proliferation.

When the osteoblasts were treated with genistein and 17β-estradiol for 72 h, genistein and 17β-estradiol resulted in the values of cell viability from 112.3 to 126.3% (Table 2). This result elucidated that genistein and 17β-estradiol also showed osteoblast proliferation. All of the hydrolysates and combinations could promote cell growth, resulting in viability values higher than 100%. Unfortunately, these data also indicated that these hydrolysates and combinations at this treatment time (72 h) usually showed weaker osteoblast proliferation than the other treatment time (48 h) (Table 2). Treatment time of 48 h was, therefore, used in later study to assess anti-apoptosis of these hydrolysates and combinations. The other results were not compared and discussed in details here. However, these combinations mostly showed stronger in osteoblast proliferation than genistein itself, or the counterpart hydrolysates alone. This fact demonstrated again that genistein could cooperate with these hydrolysates to promote greater osteoblast proliferation.

GH2, SH2, and CH2 usually showed higher osteoblast proliferative activities than their counterparts (GH1, SH1 and CH1), thus, they were selected to assess their amino acid compositions and anti-apoptosis to the osteoblasts. The hydrolysates at a dose level of 0.05 g/L mostly led to stronger osteoblast proliferation. Therefore, this dose level was used in anti-apoptosis evaluation again.

### 2.4. Cooperation between Genistein and the Hydrolysates in Osteoblast Anti-Apoptosis

**Figure 2:**
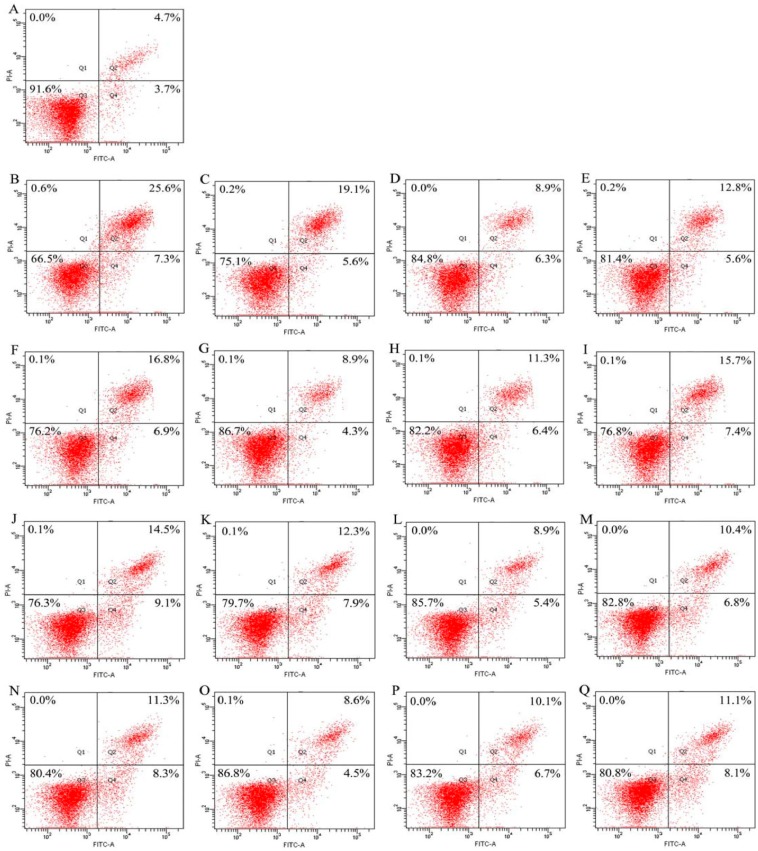
The apoptotic proportions in the osteoblasts detected using flow cytometry after apoptotic prevention by genistein (Gen), three hydrolysates, and three Gen-hydrolysate combinations. (**A**) control cells; (**B**) EP-treated cells; (**C**–**I**) the cells firstly treated by Gen, GH2, SH2, CH2, Gen-GH2, Gen-SH2, and Gen-CH2, respectively, and then treated by EP at 10 mg/L; (**J**) NaF-treated cells; (**K**–**Q**) the cells first treated by Gen, GH2, SH2, CH2, Gen-GH2, Gen-SH2, and Gen-CH2, respectively, and then treated by NaF at 40 mg/L. GH2, SH2, and CH2 were the hydrolysates generated from gelatin, de-isoflavoned soy protein, and casein with DH values of 13.7%, 13.5%, and 13.3%, respectively. The hydrolysates and Gen were used at 0.05 g/L and 2.5 μg/L, respectively. EP represents etoposide, while the labeled Q1–Q4 represent necrotic, later apoptotic, viable, and early apoptotic cells, respectively.

**Figure 3:**
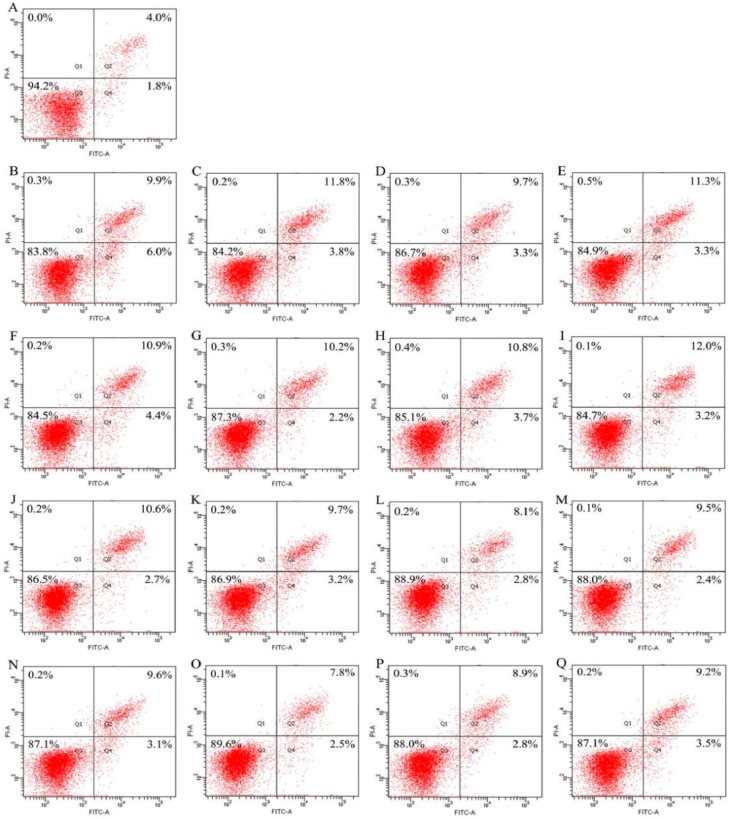
The apoptotic proportions in the osteoblasts detected using flow cytometry after apoptotic prevention by apoptotic rescue of genistein (Gen), three hydrolysates, and three Gen-hydrolysate combinations. (**A**) control cells; (**B**) EP-treated cells; (**C**–**I**) the cells firstly treated by EP at 10 mg/L, and then treated by Gen, GH2, SH2, CH2, Gen-GH2, Gen-SH2, and Gen-CH2, respectively; (**J**) NaF-treated cells; (**K**–**Q**) the cells first treated by NaF at 40 mg/L, and then treated by Gen, GH2, SH2, CH2, Gen-GH2, Gen-SH2, and Gen-CH2, respectively. GH2, SH2, and CH2 were the hydrolysates generated from gelatin, de-isoflavoned soy protein, and casein with DH values of 13.7%, 13.5%, and 13.3%, respectively. The hydrolysates and Gen were used at 0.05 g/L and 2.5 μg/L, respectively. EP represents etoposide while the labeled Q1–Q4 represent necrotic, later apoptotic, viable, and early apoptotic cells, respectively.

Both EP and NaF were used in this study to induce osteoblast apoptosis. Genistein, the three hydrolysates, and three combinations were assessed for their anti-apoptosis in modes of apoptotic prevention and rescue. The results shown in Figure 2 and Figure 3 briefly indicated that the assessed samples all had apoptotic prevention and rescue on the osteoblasts, because the treated osteoblasts had less apoptotic proportions (Q2+Q4) than the osteoblasts in the model group.

**Figure 4:**
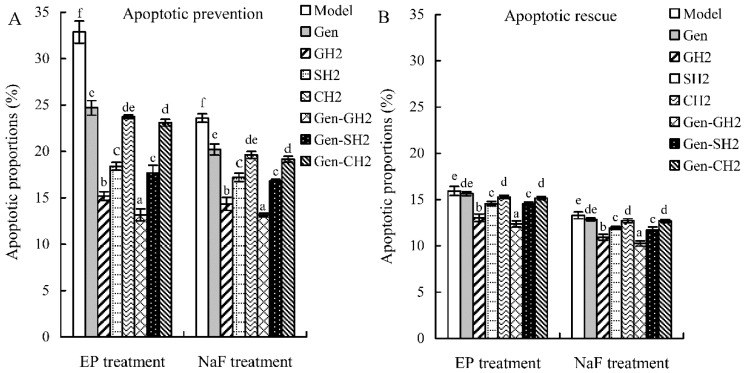
The measured apoptotic proportions (%) of the osteoblasts treated with genistein (Gen), protein hydrolysates, and Gen-hydrolysate combinations in the modes of apoptotic prevention (**A**) and rescue (**B**). All values shown represent means ± standard deviations of triplicate measurements. Different letters indicate significantly different values (*p* < 0.05) using one-way ANOVA analysis.

The results shown in Figure 2 demonstrate that genistein, the three hydrolysates, and three combinations could provide apoptotic prevention on the osteoblasts via decreasing apoptotic proportions in the EP/NaF-treated osteoblasts. The results of apoptotic prevention are summarized in Figure 4A. Apoptotic proportion of the control cells (without any treatment) was only 8.4%, whereas both EP and NaF treatments (i.e., model groups) increased apoptotic proportions to 32.9% and 23.6%, respectively. Genistein decreased apoptotic proportions to 24.7% (EP treatment) and 20.2% (NaF treatment), which evidenced that genistein provided apoptotic prevention in the osteoblasts. GH2, SH2, and CH2 showed stronger apoptotic preventive effects than genistein, as they reduced apoptotic proportions to 15.2%, 18.4%, and 23.7% (EP treatment), or 14.3%, 17.2%, and 19.6% (NaF treatment), respectively. Among three hydrolysates, GH2 demonstrated the strongest apoptotic prevention effect, revealed by the least amount of apoptotic cells. Three combinations (Gen-GH2, Gen-SH2, and Gen-CH2) reduced apoptotic proportions to 13.2%, 17.7%, and 23.1% (EP treatment), or 13.1%, 16.8%, and 19.2% (NaF treatment). Based on the comparison of these data, it is seen that the three combinations displayed stronger apoptotic prevention than the counterpart hydrolysates alone (or genistein itself), as less apoptotic cells were detected in each case. In other words, genistein cooperated with three hydrolysates, ensuring the three combinations with enhanced apoptotic prevention.

The results shown in Figure 3 elucidate that genistein, the three hydrolysates, and three combinations could also provide apoptotic rescue for the osteoblasts via decreasing apoptotic proportions in the EP/NaF-treated osteoblasts. Apoptotic proportions of the osteoblasts subjected to different treatments are also summarized in Figure 4B. Apoptotic proportion of the control cells (without any treatment) was 5.8%, while both EP and NaF treatments (i.e., model groups) enhanced apoptotic proportions to higher levels of 15.9% and 13.3%, respectively. Genistein reduced apoptotic proportions to 15.6% (EP treatment) and 12.9% (NaF treatment), indicating its weaker apoptotic rescue. GH2, SH2, and CH2 also had apoptotic rescue, as they could decrease apoptotic proportions to 13.0%, 14.6%, and 15.3% (EP treatment), or 10.9%, 11.9%, and 12.7% (NaF treatment), respectively. In the three hydrolysates, GH2 also showed the strongest apoptotic rescue, reflected by the lowest apoptotic proportions in the osteoblasts. The three combinations (Gen+GH2, Gen+SH2, and Gen+CH2) also showed weaker apoptotic rescue, as apoptotic proportions in these cases were decreased to 12.4%, 14.5%, and 15.2% (EP treatment), or 10.3%, 11.7%, and 12.7% (NaF treatment). The three combinations, thus, mostly showed somewhat stronger apoptotic rescue than the counterpart hydrolysates alone (or genistein itself). This fact indicated that genistein cooperated with the three hydrolysates, conferring the combinations with somewhat higher apoptotic rescue in the osteoblasts. However, by comparison of these data given in Figure 4A,B, it is seen that apoptotic rescue of these assayed samples was usually weaker than their apoptotic protection. This suggests that genistein, the three hydrolysates, and three combinations might exert osteoblast anti-apoptotic effects mainly through apoptotic protection, reflected by reduced proportions of early and late apoptosis.

## 3. Discussion

Isoflavones are commonly known as important phytoestrogens in plant foods because they have potential estrogenic effects in various tissues [32]. Isoflavones also have anti-bone-resorptive activity; for example, both Chinese and Japanese postmenopausal women have been surveyed with higher bone mineral density as a result of higher intake of soy protein or isoflavones [33,34]. Isoflavones are, thus, considered to preserve bone mass in postmenopausal women [35]. Both in vitro and in vivo studies have proved this beneficial effect. Genistein can promote the growth of human bone marrow mesenchymal stem cells in vitro [9] and inhibit osteoclasts formation of bone marrow cells from the methotrexate-treated rats [36]. Soy isoflavone mixtures at dose levels of 0.1–0.3 mol/L can enhance the growth of the osteoblasts isolated from skull bones of neonatal rats [37]. In the present study, genistein was also capable of promoting the growth of the osteoblasts (hFOB1.19 cells) at lower dose levels (e.g., 2.5 μg/L), evidencing its 17β-estradiol-like effect once more. The present result was consistent with those previously reported by other studies. However, higher genistein dose (e.g., 20–40 μg/L) in this study was observed to exhibit clear toxicity to the osteoblasts, resulting in lower cell viability. Similar results have been observed in other studies. For example, it has been found that genistein at 2.5–30 μmol/L (about 675–8100 μg/L) can reduce the proliferation of MG63 human osteosarcoma osteoblasts [38], or at 0.01–1 μmol/L (about 2.7–270 μg/L) it may inhibit the proliferation of rat calvaria mature osteoclasts [8]. However, genistein doses used in the present study were primarily lower than those used in these two reports.

Both gelatin and gelatin hydrolysates have been investigated for their bioactivities, for example, antihypertension and antioxidation [39,40]. Gelatin and gelatin hydrolysates have also been assessed for their effects on the bone metabolism with positive results. Bovine collagen peptides can increase the growth, differentiation, and mineralization of MC3T3-E1 cells [41]. Shark gelatin is capable of increasing bone mineral density of the ovariectomized rats through oral administration [42], while chum salmon skin gelatin hydrolysates can promote osteoblast growth and antagonize NaF-induced osteoblast apoptosis [23]. In addition, other hydrolysates, such as those from soy protein and casein, have also been reported with beneficial effects on the bone metabolism. Soy protein and casein hydrolysates can accelerate bone turnover with bone formation exceeding resorption and, thus, increase bone mineral density [43], promote the growth of the osteoblasts, and also decrease the induced osteoblast apoptosis [26]. These mentioned results support that the protein hydrolysates prepared in this study could promote proliferation and inhibit apoptosis of the osteoblasts. The present results also indicated that GH2 had the highest activities compared to other hydrolysates. This may be partially caused by typical amino acid compositions of GH2, as GH2 contained more Ala, Gly, Pro, and especially 4-Hyp (Table 1). It is well known that type I collagen is a major component of the bone organic matrix, and can provide a stable template for bone mineralization [44]. Some important biological functions of the osteoblasts (e.g., proliferation and differentiation) depend on collagen [41]. 4-Hyp is a specific amino acid in collagen [45], but is not an element in casein and soy protein. More importantly, both Pro and 4-Hyp have been observed to be helpful to bone metabolism [46]. It has been evidenced that both Pro and Hyp regulate the differentiation of chondrocytes into their mineralized form, and inhibit phosphorus-induced degradation of mice cartilage including thinning of articular cartilage layer and loss of chondrocytes [47]. Both Pro and Hyp are able to enhance hyaluronic acid synthesis in cultured synovial cells [48]. All of these mentioned results point out a fact; namely, it was most likely that both Pro and 4-Hyp made positive contributions to the higher effects of the gelatin hydrolysates than other protein hydrolysates used in this study.

Prevention of osteoporosis through a dietary approach is important and practical. Several compounds (e.g., omega-3 fatty acids and calcium) have been studied for their prevention separately [32]. However, foods consumed per day contain many compounds instead of a single one. Therefore, a mixture composed of two (or more) compounds should be studied for its preventive effect. Combinations of isoflavones and other food components have been studied for their effects on bone health. When isoflavones are mixed with V_D3_, there exist enhanced effects in terms of increasing bone mineral density, preosteoblast proliferation, and collagen type I expression [8]. A combination of isoflavones and resistant starch is capable of preventing ovariectomy-induced decline in trabecular bone mineral density, altering immune status in the bone marrow, and resulting in attenuated bone resorption in ovariectomized mice [49]. Bovine growth hormone and 1,25(OH)_2_V_D3_, separately or in combination, can all inhibit apoptosis and enhance growth of UMR 106 osteoblast-like cells [50]. Potential cooperative effects of genistein (or isoflavones) and protein hydrolysates on the osteoblasts or bone are important and interesting issues. The present study showed that the assessed genistein-hydrolysate (especially GH2) combination had higher proliferation and anti-apoptosis than genistein itself, or of the used hydrolysates alone. A similar cooperative effect has been found in a combination of isoflavones and milk basic protein, as this mixture is observed to prevent bone loss of the female mice better in comparison to the single treatment [51]. This finding agrees that genistein could cooperate with these hydrolysates for higher activities. In a reported in vivo study [52], if a mixture composed of soy protein and isoflavones is used to feed female mice for 14 days, the mixture is more able to increase bone mineral density than soy protein or isoflavones. However, soy protein used in this reported research contains naturally-occurring isoflavones (i.e., it is not a de-isoflavoned protein product); therefore, the reported result might only reflect the in vivo effect of soy protein with various isoflavone contents on bone mineral density. To reflect practical activities of soy protein hydrolysates, it was necessary that soy protein used in this study was de-isoflavoned using an ethanol solution. When de-isoflavoned soy protein was used to generate two hydrolysates (SH1 and SH2), both SH1 and SH2 would be free of the naturally-occurring isoflavones. This pretreatment of soy protein ensured this study to assess the cooperative effects between genistein and two soy protein hydrolysates in two activities.

Apoptosis is a crucial determinant of the life span of the osteoblasts in bone-forming function [53]. The initiation of apoptosis typically leads to the activation of caspases, which can cleave different proteins to release proapoptotic protein fragments [54,55,56]. Nevertheless, the activity of these proapoptotic protein fragments can be impaired by their selective degradation via the N-end rule pathway [55,57,58]. In other words, the N-end rule pathway relates regulation of in vivo half-life of proteins to the identity of its N-terminal residue, which is involved in the anti-apoptotic function [59]. A finding from Eldeeb and Fahlman [57] reveals that activation of apoptotic pathways leads to the caspase cleavage of the Lyn tyrosine kinase to generate the N-terminal truncated LynΔN. In addition, selective degradation of a caspase product via the N-end rule pathway is modulated by phosphorylation [58]. In this work, the anti-apoptotic effects of genistein, three protein hydrolysates, and three combinations on human osteoblasts maybe attributed to their regulatory role in expression of apoptosis-related molecules via the N-end rule pathway. This study provides preliminary evidence about the cooperative effects between genistein and protein hydrolysates in proliferation and anti-apoptosis of the osteoblasts, and, thus, gives further guidance in the development of functional ingredients for osteoporosis prevention. However, the related molecular mechanisms responsible for the proliferation, and especially anti-apoptosis of the protein hydrolysates and genistein-hydrolysate combinations, remain to be further investigated through the detection of gene and protein expressions in the osteoblasts.

## 4. Materials and Methods

### 4.1. Chemicals and Reagents

Genistein (>98% purity) was bought from Shanghai u-Sea Biotechnology Co. Ltd. (Shanghai, China). Chum salmon skin gelatin and casein were purchased from Xueyang Gelatin Co. Ltd. (Cangzhou, Hebei, China) and Beijing Aoboxing Biotechnologies Inc. (Beijing, China), respectively. Soy protein isolate was prepared from defatted soy flour as previously described [60]. Papain was purchased from Sinopharm Chemical Reagent Co. Ltd. (Shanghai, China). Fetal bovine serum (FBS) was obtained from Wisent Inc. (Montreal, QC, Canada). Activated charcoal, etoposide (EP), sodium fluoride (NaF), G418, and DMEM:Ham’s F12 (1:1) medium were purchased from Sigma Chemical Co. (St. Louis, MO, USA). Cell Counting Kit-8 (CCK-8) was purchased from Dojindo Molecular Technologies, Inc. (Kyushu, Japan). Annexin V-FITC/PI Apoptosis Detection Kit was bought from Beyotime Institute of Biotechnology. (Shanghai, China). The following reagents were purchased from Solarbio Science and Technology Co. Ltd. (Beijing, China): dextran T-70, trypsin-EDTA, phosphate-buffered saline (PBS), and dimethyl sulfoxide (DMSO). The water used in this study was ultrapure water generated from Milli-Q Plus water purification system (Millipore Corporation, New York, NY, USA). Other chemicals used were analytical grade.

The activated charcoal/dextran T-70 treated with the FBS was prepared by a modified method of Eckert and Katzenellenbogen [61]. In brief, 250 mg activated charcoal and 25 mg dextran T-70 were mixed with 100 mL FBS, followed by an incubation of 45 min at 55 °C. The insoluble particulates in the FBS were removed by centrifugation at 9000× *g* for 15 min. The above steps were repeated three times. The FBS was filtered through a 0.22 μm filter to obtain the sterilized FBS and then used in experiments.

### 4.2. Preparation of De-Isoflavoned Soy Protein and Protein Hydrolysates

An ethanol extraction method was used to prepare de-isoflavoned soy protein. The prepared soy protein isolate of 100 g was dispersed in a 75% ethanol solution of 500 mL to extract these naturally-occurring isoflavones. The mixture was placed in a water bath operated at a temperature of 30 °C, stirred continually for 1 h and then filtered to collect residual materials. The filtrate (containing isoflavones) was measured for its absorbance at 260 nm (the typical absorption wavelength of isoflavones) using a spectrophotometer (UV-2401PC, Shimadzu, Tokyo, Japan). The residual materials were then retreated with the ethanol solution five times, until the measured absorbance of the filtrate was near zero. The residual materials were considered to be negligible in those naturally-occurring isoflavones, and then lyophilized to obtain de-isoflavoned soy protein.

Gelatin, de-isoflavoned soy protein, and casein of 5 g on a dry basis were all dispersed separately in 100 mL of water, and then adjusted to pH 6.0 via adding 1 mol/L HCl or NaOH. The generated protein solutions were incubated with papain of 3 kU/g protein in a water bath of 60 °C to trigger protein hydrolysis. At different hydrolysis times, the hydrolyzed protein solutions of 15 mL were separated and placed in other vessels, followed immediately by a heating treatment for 15 min at a boiling water to inactivate papain. The hydrolyzed protein solutions were cooled to ambient temperature and centrifuged at 11,000× *g* for 20 min. The supernatants were collected to obtain protein hydrolysates, which were then measured for their DH values. Afterwards, each of three proteins was further hydrolyzed with a selected hydrolysis time to prepare the protein hydrolysates with two different DH values (around 7.5% and 13.5%). These generated hydrolysates were designed as respective GH1-2, SH1-2, and CH1-2, lyophilized, and stored at 20 °C for later use.

### 4.3. Cell Line and Culture Conditions

A human fetal osteoblast cell line (hFOB 1.19 cells) was provided by Cell Bank of the Chinese Academy of Sciences (Shanghai, China). In the presence of 0.6 mg/mL neomycin G418, the osteoblasts were obtained from the limb tissue of a spontaneous fetal miscarriage with the temperature-sensitive expression vector pUCSVisA58. The osteoblasts were recommended to be inoculated with the DMEM:Ham’s F-12 (1:1) medium without phenol red supplemented with 10% FBS (*v/v*) and 0.3 mg/mL neomycin G418. The osteoblasts were cultured at 34 °C with 5% CO_2_ in a humidified atmosphere as recommended by the cell supplier.

### 4.4. In Vitro Effect of Genistein on the Osteoblasts

This evaluation was carried out by a Cell Counting Kit-8 (CCK-8) method as previously described [26]. The WST-8 reagent contained in the CCK-8 can be reduced into an orange water-soluble formazan dye by dehydrogenase in living cells [62]. The amount of the formazan dye is proportional to the numbers of living cells.

The cells were seeded onto 96-well plates at 5 × 10^3^ cells per well, and incubated at 34 °C to allow their adherence in normal culture medium. After a culture time of 24 h, the cells were starved with the culture medium containing 0.5% FBS overnight, and incubated with genistein of 1.25–40 μg/L in a total volume of 200 μL normal culture medium for 48 h. After the incubation, the medium was discarded. CCK-8 solution (10 μL CCK-8 in 100 μL normal culture medium) of 110 μL was added to each well. After another incubation of 4 h, the absorbance (OD value) of each well was detected at a wavelength of 450 nm using a microplate reader (Bio Rad Laboratories, Hercules, CA, USA), and used to calculate cell viability. The vehicle-treated cells were served as 100% viable.

### 4.5. Assay of Cell Proliferation

The cells were seeded onto 96-well plates (5 × 10^3^ per well). Genistein (2.5 μg/L), the hydrolysates (0.02–0.1 g/L), 17β-estradiol (positive control, 10^−8^ mol/L), and genistein-hydrolysate combinations (genistein 2.5 μg/L plus hydrolysates 0.02–0.1 g/L) in 200 μL normal culture medium were added to the wells, respectively. Both genistein and 17β-estradiol were firstly dissolved in DMSO, and made up with the culture medium so that the final DMSO concentration was less than 0.1% (*v/v*). The cells were then incubated at 34 °C for 48 and 72 h, respectively. Afterwards, the CCK-8 solution of 110 μL was added to each well, followed by same cell incubation and absorbance detection in Section 4.4. The vehicle-treated cells were taken as 100% viable. Cell viability was calculated to reflect cell proliferation.

### 4.6. Apoptosis Assay

Both Annexin V-FITC and propidium iodide (PI) as fluorescent dyes were used in this study to detect the induced osteoblast apoptosis. Annexin V labeled by FITC can identify these early and late apoptosis cells, while late apoptosis and necrotic cells can be stained by PI [23,26].

In the mode of apoptotic prevention, the cells were seeded onto six-well plates, and incubated with genistein (2.5 μg/L), three hydrolysates (GH2, SH2, and CH2, 0.05 g/L) and three combinations with genistein (2.5 μg/L) and one of three hydrolysates (GH2, SH2, and CH2, 0.05 g/L) for 48 h, respectively. Afterwards, the cells were treated for 24 h with one of the two pro-apoptotic agents, EP (10 mg/L) and NaF (40 mg/L), as previously described [23,26]. In the mode of apoptotic rescue, the cells were seeded onto six-well plates, treated by one of the two pro-apoptotic reagents for 24 h, and then cultivated for 48 h with genistein (2.5 μg/L), the three hydrolysates (GH2, SH2, and CH2, 0.05 g/L) and three combinations, respectively. The cells without any treatment served as the control. After these treatments, the cells were resuspended by trypsin treatment, and washed twice with PBS solution (0.1 mol/L, pH 7.2) of 1 mL. An Annexin V-FITC/PI Apoptosis Detection Kit was used to detect the cells according to the manufacturer’s protocol. In brief, the harvested cells were resuspended in 500 μL Annexin V-FITC blinding buffer, and stained by 5 μL of the Annexin V-FITC and 10 μL of PI. The cells were kept in the dark at ambient temperature for 20 min, and then were detected using flow cytometry (FACS Calibur, Becton Dickson, San Jose, CA, USA) to obtain the proportions of intact, apoptotic, and necrotic cells as previously described [63].

### 4.7. Chemical Analyses

Nitrogen contents of the analyzed samples were assessed by the Kjeldahl method [64] and multiplied by respective conversion factors of 5.55, 6.25, and 6.38 to obtain respective protein contents of gelatin, de-isoflavoned soy protein, and casein (or respective hydrolysates).

Free amino groups were assayed by the o-phthaldialdehyde (OPA) method [65] with slight modifications. The assay was performed by adding hydrolysate solutions (or standard leucine solutions) of 3 mL to the OPA reagent of 3 mL. The absorbance of the mixed solution was measured by the spectrophotometer at 340 nm after a reaction time of 5 min. l-Leucine solutions of 6–36 μg/mL were used to generate the standard curve for this measurement. The DH value of each hydrolysate was calculated as DH (%) = (*h*/*h_tot_*) × 100, where *h* was the cleaved numbers of peptide bonds per unit weight, and *h_tot_* was the total numbers of peptide bonds per unit weight. The suggested *h_tot_* values are 11.1, 7.8, and 8.2 meq/g protein for gelatin, soy protein, and casein [66], respectively.

The 17 amino acids (Ala, Arg, Asp, Cys, Glu, Gly, His, Ile, Leu, Lys, Met, Phe, Pro, Ser, Thr, Tyr, and Val) of GH2, SH2, and CH2 were analyzed by Heilongjiang Provincial Academy of Agricultural Sciences using an automated amino acid analyzer (L-8800, Hitachi Co., Ltd., Tokyo, Japan). 4-Hydroxyproline (4-Hyp) was analyzed using a method reported by Bergman and Loxley [67], whilst Trp was assayed as previously described [68]. Contents of the 19 amino acids were all reported as g/kg protein.

### 4.8. Statistical Analysis

In this study, the reported data were expressed as means or means ± standard deviations from at least three independent preparations or evaluations. Statistical analysis was performed with the aid of SPSS software version 16.0 (SPSS Inc., Chicago, IL, USA), using one-way ANOVA with Duncan’s multiple range tests. The statistical significance was set at *p* < 0.05.

## 5. Conclusions

Genistein, three protein hydrolysates, and three genistein-hydrolysate combinations could promote the growth of the human fetal osteoblasts in vitro and, in particular, were able to prevent or to lessen the extent of the EP/NaF-induced osteoblast apoptosis. Moreover, the combination treatments led to enhanced proliferation and anti-apoptosis in osteoblasts compared to genistein and their counterpart hydrolysates alone, indicating that cooperative effects existed between genistein and these hydrolysates (especially gelatin hydrolysates) in osteoblast proliferation and anti-apoptosis. This fact revealed that the cooperative effect was important and desirable to the functional foods with bone health functions. It is, thus, suggested that potential cooperative effects between other isoflavones and proteins/peptides on anti-apoptosis in the osteoblasts should be further investigated to better understand bone health-promoting functions of isoflavones and proteins/peptides.

## Figures and Tables

**Table 1:** Amino acid compositions (g/kg protein) of three hydrolysates.

Amino Acids	Hydrolysates			Amino Acids	Hydrolysates		
	GH2	SH2	CH2		GH2	SH2	CH2
Ala	100.1	39.0	31.2	Lys	37.7	73.7	80.2
Arg	90.0	87.6	34.8	Met	8.1	14.1	32.0
Asp	54.1	128.1	77.2	Phe	22.6	52.1	51.2
Cys	ND	14.2	ND	Pro	146.4	61.2	109.2
Glu	112.9	245.5	229.5	Ser	34.7	59.8	59.8
Gly	266.1	47.1	21.8	Thr	19.2	39.4	46.9
His	6.2	25.5	27.3	Trp	ND	12.5	14.6
4-Hyp	110.7	ND	ND	Tyr	0.5	34.8	56.7
Ile	16.2	46.8	55.1	Val	31.5	44.5	67.3

Note: GH2, SH2, and CH2 are the hydrolysates generated from gelatin, de-isoflavoned soy protein, and casein with DH values of 13.7%, 13.5%, and 13.3%, respectively. ND, not detectable.

**Table 2:** Cell viability of the osteoblasts treated with genistein (Gen), protein hydrolysates, and Gen-hydrolysate combinations for 48 or 72 h.

Groups	Dose Levels	Treatment Times	
		48 h	72 h
Gen	2.5 μg/L	109.4 ± 4.1	112.3 ± 6.0
17β-Estradiol	10^−8^ mol/L	134.2 ± 2.9	126.3 ± 3.5
GH1	0.02 g/L	108.4 ± 2.6	109.4 ± 1.6
GH1	0.05 g/L	121.8 ± 2.5	117.9 ± 2.2
GH1	0.1 g/L	114.6 ± 1.9	113.2 ± 1.4
Gen-GH1	2.5 μg/L+ 0.02 g/L	113.8 ± 1.9	115.1 ± 2.2
Gen-GH1	2.5 μg/L + 0.05 g/L	127.2 ± 2.6	123.1 ± 2.8
Gen-GH1	2.5 μg/L + 0.1 g/L	119.2 ± 1.3	117.9 ± 2.9
GH2	0.02 g/L	122.7 ± 2.8	110.0 ± 1.4
GH2	0.05 g/L	131.1 ± 4.3	125.3 ± 2.1
GH2	0.1 g/L	124.0 ± 2.0	116.7 ± 1.4
Gen-GH2	2.5 μg/L + 0.02 g/L	125.3 ± 3.4	115.8 ± 2.1
Gen-GH2	2.5 μg/L + 0.05 g/L	140.9 ± 1.3	131.2 ± 4.1
Gen-GH2	2.5 μg/L + 0.1 g/L	130.2 ± 2.3	122.2 ± 3.6
SH1	0.02 g/L	113.2 ± 5.7	115.8 ± 6.2
SH1	0.05 g/L	110.4 ± 4.1	110.9 ± 2.6
SH1	0.1 g/L	104.6 ± 6.0	104.6 ± 0.8
Gen-SH1	2.5 μg/L + 0.02 g/L	114.6 ± 4.5	118.7 ± 4.4
Gen-SH1	2.5 μg/L + 0.05 g/L	111.1 ± 4.4	114.8 ± 4.7
Gen-SH1	2.5 μg/L + 0.1 g/L	104.8 ± 3.2	108.7 ± 2.2
SH2	0.02 g/L	119.8 ± 5.7	107.7 ± 3.3
SH2	0.05 g/L	121.3 ± 2.4	110.6 ± 5.6
SH2	0.1 g/L	118.0 ± 1.2	104.4 ± 2.2
Gen-SH2	2.5 μg/L + 0.02 g/L	121.1 ± 1.2	110.9 ± 3.3
Gen-SH2	2.5 μg/L + 0.05 g/L	123.1 ± 0.9	112.3 ± 3.4
Gen-SH2	2.5 μg/L + 0.1 g/L	119.6 ± 1.8	107.1 ± 3.9
CH1	0.02 g/L	107.6 ± 4.9	106.6 ± 5.1
CH1	0.05 g/L	108.6 ± 4.4	105.9 ± 5.8
CH1	0.1 g/L	102.9 ± 6.6	105.1 ± 5.6
Gen-CH1	2.5 μg/L + 0.02 g/L	108.6 ± 3.3	108.1 ± 4.4
Gen-CH1	2.5 μg/L + 0.05 g/L	110.5 ± 2.9	107.4 ± 1.3
Gen-CH1	2.5 μg/L + 0.1 g/L	101.9 ± 4.9	104.4 ± 4.6
CH2	0.02 g/L	107.9 ± 3.9	108.0 ± 3.3
CH2	0.05 g/L	112.4 ± 5.1	114.6 ± 4.6
CH2	0.1 g/L	105.6 ± 1.9	113.1 ± 6.7
Gen-CH2	2.5 μg/L + 0.02 g/L	107.9 ± 7.0	107.3 ± 5.8
Gen-CH2	2.5 μg/L + 0.05 g/L	114.6 ± 5.1	116.8 ± 5.5
Gen-CH2	2.5 μg/L + 0.1 g/L	106.7 ± 3.4	113.9 ± 3.8

Note: GH1 and GH2 were gelatin hydrolysates with DH values of 7.7% and 13.7%; SH1 and SH2 were de-isoflavoned soy protein hydrolysates and with DH values of 7.6% and 13.5%; CH1 and CH2 were casein hydrolysates with DH values of 7.3% and 13.3%, respectively.

## Abbreviations

Gen	genistein
CH	casein hydrolysate
GH	gelatin hydrolysate
SH	soy protein hydrolysate
DH	degree of hydrolysis
CCK-8	cell counting kit-8
DMSO	dimethyl sulfoxide
E	17β-estradiol
WST-8	2-(2-methoxy-4-nitrophenyl)-3-(4-nitrophenyl)-5-(2,4-disulfophenyl)-2*H*-tetrazolium, monosodium salt
EDTA	ethylenediamine tetra-acetic acid
EP	etoposide
FACS	fluorescence-activated cell sorting
FBS	fetal bovine serum
hFOB 1.19 cells	human fetal osteoblastic cells
OPA	o-phthaldialdehyde
PBS	phosphate-buffered saline
